# Resting-state fMRI dynamic functional network in young children with Tourette syndrome

**DOI:** 10.3389/fneur.2026.1758013

**Published:** 2026-03-20

**Authors:** Haode Wang, Yue Liu, Chen Zhang, Jingtao Lian, Lei Yang, Yun Peng

**Affiliations:** 1 China Academy of Information and Communications Technology, Beijing, China; 2 NMOE Key Laboratory of Major Diseases in Children, Department of Radiology, Beijing Children's Hospital, Capital Medical University, National Center for Children’s Health, Beijing, China; 3 Department of Radiology, Children’s Hospital Affiliated to Zhengzhou University, Henan Children’s Hospital Zhengzhou Children’s Hospital, Zhengzhou, China

**Keywords:** dynamic functional network, functional magnetic resonance imaging, insula, resting-state, Tourette syndrome

## Abstract

**Introduction:**

Tourette syndrome (TS) is a neurodevelopmental disorder characterized by involuntary motor and phonic tics, with diagnosis often delayed due to the 1-year symptom duration criterion. This study aimed to explore early neuroimaging biomarkers of TS in young children, by investigating spatiotemporal alterations in dynamic brain network connectivity in 4-6-year-old children with TS.

**Methods:**

This retrospective case-control study collected resting-state functional magnetic resonance imaging (fMRI) data from 24 children aged 4-6 years, including 12 drug-naive TS patients and 12 matched healthy controls (N group). Group Independent Component Analysis (GICA) and Independent Vector Analysis (IVA) were used to assess group differences in temporal and spatial dynamic functional network connectivity (FNC), respectively. Correlations between these connectivity alterations and Yale Global Tic Severity Scale (YGTSS) scores as well as disease duration were analyzed.

**Results:**

Statistically significant between-group differences were found in temporal dynamic FNC (*p* < 0.05), with marginal differences observed in spatial dynamic connectivity (*p* < 0.1), primarily involving the ventral default mode network (VDMN), primary visual network (PVN), and precuneus network (PCN). The N group showed a wider range and higher strength of FNC values, while the TS group exhibited abnormally enhanced connectivity in the insula region, which was positively correlated with disease duration.

**Discussion:**

This study revealed abnormal temporal and spatial dynamic brain network connectivity in young children at the early stage of TS, particularly in insula-related circuits. These findings provide novel insights into the early neuropathological mechanisms of TS and support the potential of dynamic FNC metrics as early imaging biomarkers for TS in young children.

## 1 Introduction

Tourette syndrome (TS) is a neurodevelopmental disorder causing involuntary muscle movements and sounds termed tics. TS usually begins in childhood, with a typical age of onset between 5 and 7 years and an incidence ranging from 0.5 to 0.8% (1). Despite its low incidence, TS may seriously impact the future life of patients. For example, in a follow-up study of TS, 79% of patients had at least some tics, 40% reported some level of social impairment, and 20% were either unemployed, disabled or financially dependent on families (2). Another study revealed significantly enhanced substance abuse and consequences including substance-related death in TS cases (3). Meanwhile, a significantly increased risk of traumatic brain injury was found in TS patients compared with controls (4).

To date, TS diagnosis mostly depends on the qualitative description of symptoms and the disorder could be misdiagnosed because of varying presentation (5) or the interference of other comorbidities, causing delayed treatment and disease aggravation. Early diagnosis and treatment of TS could help alleviate the disorder and avoid serious complications. Crucially, a timely diagnosis allows for the monitoring of the onset of common comorbidities such as attention-deficit/hyperactivity disorder (ADHD), obsessive-compulsive disorder (OCD), and anxiety, preventing the child from facing the burden of multiple undiagnosed issues simultaneously. Therefore, developing early and accurate diagnostic biomarkers is of great importance. Recent studies suggested many brain areas, including primary sensorimotor cortex, supplementary motor area (SMA), premotor cortex, cingulate motor cortex, lateral premotor cortex, basal ganglia, inferior parietal cortex, and cerebellum, are structurally and functionally altered and/or function in brain magnetic resonance imaging (MRI) of TS cases (6, 7). Specifically, Ramkiran et al. (8) applied graph measures to resting-state (RS)-fMRI scans of adults with TS and assessed the functional properties of different portions of cortico-basal ganglia-cerebellar networks. The findings suggested disrupted interoceptive mechanisms and impaired brain maturation, as well as a shift toward excitatory neurotransmission in TS. Another RS study found altered functional connectivity (FC) in frontoparietal areas in TS patients (9, 10), indicating an immature functional brain organization. These areas are also involved in multitasking and are thought to play inhibitory roles during inappropriate responses (11).

Previous RS-fMRI investigations have primarily relied on static descriptions of FC, and the resulting characterization ultimately represented a time-averaging phenomenon. In contrast, the human brain is seldom at rest, exhibiting correlated and spontaneous activity dynamics (12). Even in the resting state, FC may change in time, and conventional analysis could not fully assess its properties. For TS research, dynamic FC analyses provide a more sensitive framework than static approaches by capturing short-lived brain network fluctuations and revealing subtle, state-specific abnormalities. These abnormalities, possibly related to the intrinsic temporal variability of tic expression, are often masked by time-averaged FC. For instance, children with TS exhibit altered dwell times and temporal variability in network states and nodal efficiency, which correlate with tic severity (13–15). Moreover, dynamic FC metrics correlate more strongly with clinical measures of tic severity and disease progression than static FC, offering a more sensitive tool for assessing symptom severity and tracking treatment effects (16, 17).

This advantage of dynamic analysis is supported by recent brain function studies on psychiatric disorders. For example, research in ADHD and affective disorders has demonstrated that dFC provides superior sensitivity to transient brain-state changes and better correlates with cognitive flexibility and emotional regulation than static FC (18–20). Additionally, recent reviews have highlighted that the temporal stability and switching properties of dynamic networks represent key indicators of brain functional integrity, further emphasizing the superiority of dynamic over static approaches for understanding complex brain dynamics (21, 22). Collectively, these findings suggest that qualitative changes in FC metrics over time and space might provide greater insights into TS-indued alterations of brain networks.

The typical onset age of TS is around 5–7 years. However, according to the US Child Mental Health Monitoring Report, 0.3% of children and adolescents have been diagnosed with TS, with a minimum age of 3 years (23). Recent reviews highlight that TS is a childhood-onset neurodevelopmental disorder with a broad clinical spectrum, typically manifesting between 4 and 6 years of age (24). Moreover, to be diagnosed with TS, an individual must have had tics for at least 1 year. However, it was suggested that the current 12-month cutoff for chronicity may be arbitrary (25). These reports jointly suggest younger children are also likely to have TS.

To examine the early changes of the spatiotemporal dynamic brain network in clinical TS, 4–6-year-old TS patients and healthy subjects’ fMRI data have been analyzed. Through temporal and spatial dynamic Functional Network Connectivity (FNC) analysis (implemented via Group Independent Component Analysis (GICA) and Independent Vector Analysis (IVA), respectively), the potential abnormalities in the dynamic brain network were comprehensively examined, and correlations between the abnormality and clinical symptoms (YGTSS score and disease duration) were explored.

## 2 Materials and methods

### 2.1 Subjects and diagnostic scales

Twelve TS patients (TS group; 4.93 ± 0.60 years, 2 females and 10 males) and 12 healthy controls (N group; age 5.36 ± 0.95 years, 2 females and 10 males) were recruited from Outpatient Clinic, Beijing Children’s Hospital, Capital Medical University between July 2012 and November 2020. As drugs can significantly affect the structure and function of the central nervous system in TS (26), only drug-naïve subjects were included. The American Psychiatric Association’s Diagnostic and Statistical Manual of Mental Disorders (Fifth Edition; DSM-5) was utilized to diagnose tic disorders. OCD was diagnosed by the clinical interview method and the Children’s Yale-Brown Obsessive-Compulsive Scale (CY-BOCS) (27). The Devon Wender-Utah Rating Scale (WURS-k) was employed to diagnose Attention deficit and hyperactivity disorder (ADHD) (28). Patients meeting criteria for autism spectrum disorder (ASD), OCD, ADHD or other learning disabilities, depression or anxiety were excluded. Tic severity was scored with the Yale Global Tic Severity Scale (YGTSS) (29), ranging from 10 to 79 (44.66 ± 17.96). Disease duration in the selected patients were 1 month to 1 year (4.09 ± 3.02 months). This study was approved by the Beijing Children’s Hospital Review Board. Signed informed consent was obtained from all parents/guardians following the Declaration of Helsinki.

### 2.2 Data acquisition and preprocessing

RS data were acquired with a 3.0 T MRI scanner (Gyroscan Interma Nova, Philips, Netherlands) in the Radiology Department of Beijing Children’s Hospital. Single-echo planar imaging was performed with the following parameters: repetition time (TR), 2000 ms; echo time (TE), 39 ms; slice thickness, 4 mm; slit, 1 mm; flip angle, 90°; matrix, 64 × 64; field of view (FOV), 240 mm × 240 mm. To minimize motion artifacts common in young children, chloral hydrate was utilized in both groups to reduce motion artifacts. Independent high-resolution 3D structural images were acquired in the T1-weighted MP-RAGE sequences: 192 axial slices; TR, 1160 ms; TE, 4.21 ms; inversion time, 600 ms; slice thickness, 0.9 mm; no gaps; flip angle, 15°; matrix = 512 × 512; Field of View, 256 mm × 256 mm. fMRI data were preprocessed with the SPM software (version 12).^1^ Realignment was implemented first considering the sequential acquisition. Images were recorded to the first image in the series. Then slice-timing correction was performed using the middle slice as the reference as the reference. A single T1-weighted image was co-recorded with the corrected average functional image. Then functional images were spatially normalized using parameters estimated by nonlinearly registering gray and white matter images into the Montreal Neurological Institute (MNI) space. The resampled voxel was 3 mm × 3 mm × 3 mm. Finally, the volumes were spatially smoothed with a Gaussian kernel of 6 mm fullwidth at half maximum.

### 2.3 Data analysis

It was suggested that even at rest, FC might change with time and space, and conventional analysis could not fully assess its properties. Dynamic studies have been carried out not only in the temporal domain, but also in the spatial domain. The current study employed GIFT^2^ to implement component extraction in both time and space domains of fMRI data.

The current study employed GIFT to implement component extraction. We adopted a complementary approach using both GICA and IVA with Gaussian-Laplacian source priors (IVA-GL). GICA was selected to establish a consistent group-level spatial reference, facilitating the direct comparison of temporal dynamic FNC across subjects. In contrast, IVA-GL was specifically chosen to capture inter-subject spatial variability and spatial dynamics, as it effectively handles spatial heterogeneity while preserving component correspondence.

#### 2.3.1 Temporal dynamic component extraction and analysis

##### 2.3.1.1 TDC extraction

The time-domain concatenation GICA method (30) was employed to decompose the obtained data into different signal components. To achieve a sufficient “functional parcellation” of refined cortical and subcortical components corresponding to well-known anatomical and functional segmentations, 100 divisions of TDCs were assessed (31). Data in all subjects were dimensionally reduced and spliced, and the Infomax Independent Component Analysis (ICA) algorithm was utilized to perform the calculation for 100 times in ICASSO, among which the smallest cluster number was 80 to ensure data stability. The specific procedures were previously described (32). The derived images for all subjects were converted into the Nifti format to label TDCs based on RSN templates in the GIFT toolbox (icatb/icatb_templates/RSN.zip). The parceled 100 TDCs may contain noise to be removed using two criteria. Firstly, the cross correlation between the generated TDCs and RSN templates was determined, and TDCs with a correlation coefficient below 0.2 were considered artifacts (33). Secondly, the spatial distribution of the TDC and its temporal/spectral features should have a high spatial overlap with the gray matter and a low overlap with other tissues (34).

##### 2.3.1.2 TDC analysis

Temporal static FNC analysis was then carried out. The entire time series was employed to estimate the Pearson correlation coefficient for each effective TDC. This has been widely used in previous studies and would not be repeated here.

Finally, temporal dynamic FNC analysis was then performed. The most commonly employed strategy for examining dynamics in RS FNC is the sliding window approach. This process results in the quantitation of the time-varying behavior of the chosen metric over the duration of the scan (35). All time windows were classified (usually clustered by k-means) to obtain the distribution status of network connections over the entire time series. In this work, the window width was 18 TRs, and the step was 1TRs, which was the minimum window size with significant correlations (36). Before classification by k-means, all time windows were down-sampled for each subject and only those with the largest variance of local FC were selected for pre-clustering. As a result, the optimal number of classifications was provided, and all data were classified based on the recommended number.

#### 2.3.2 Spatial dynamic component extraction and analysis

##### 2.3.2.1 SDC extraction

Except for TDCs, fluctuations associated with SDC over time were of interest as well. SDCs were extracted by IVA (37), an extension of the ICA to multiple datasets that can capture spatial variations.

**Figure 1:**
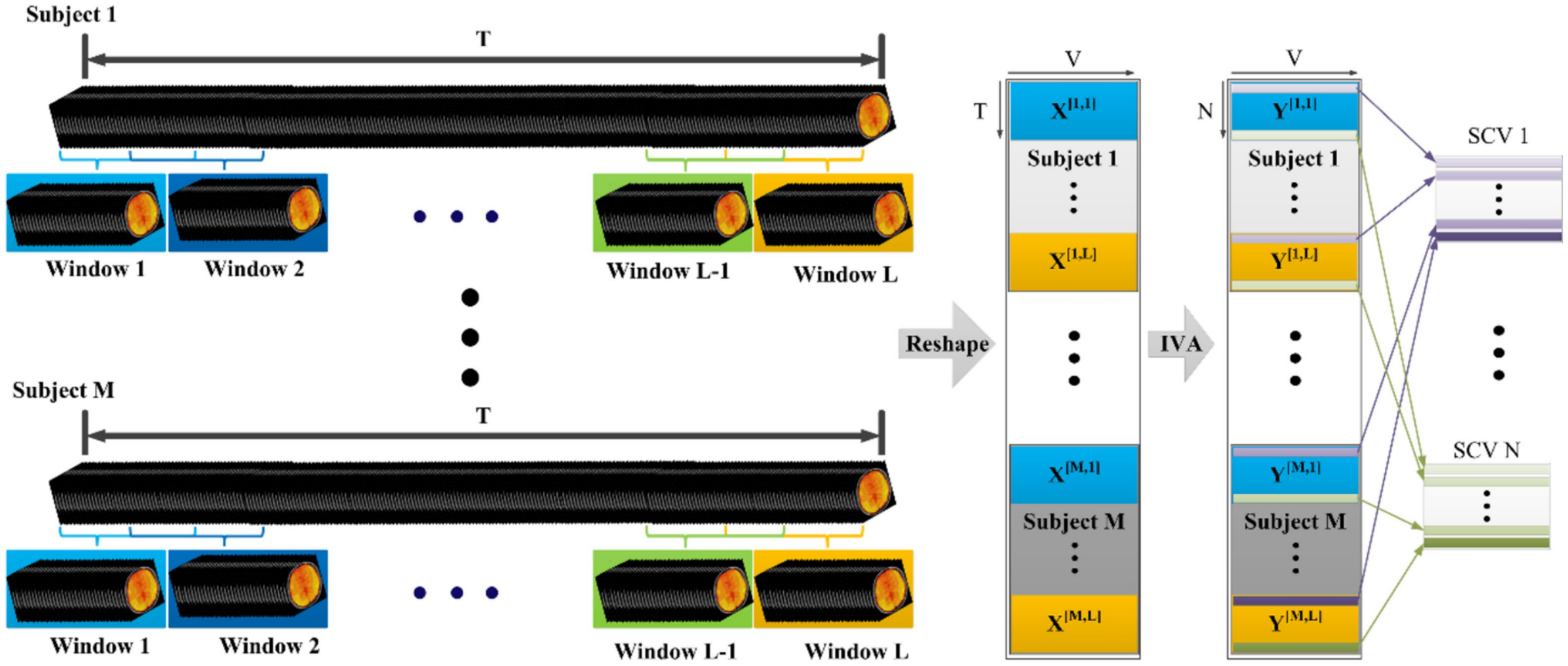
Illustration of the SDC extraction workflow. The left panel demonstrates the data segmentation process, where the fMRI time series for each subject (*m* = 1,…, M) is divided into *L* overlapping windows. The right panel details the computation process for SDC extraction: data within each time window are reshaped into an observation matrix X^[*m,l*]^ of size *T* × *V*. These matrices, collected from all *M* subjects and *L*, serve as input for the joint IVA algorithm. The algorithm estimates the matrices to yield independent source matrices Y^[*m,l*]^ (size *N* × *V*). Arrows on the far right illustrate the aggregation of the *n*-th component across all subjects and windows to form the Source Component Vector (SCV). This framework enables IVA to capture spatial variations (SDCs) across subjects and time windows while preserving component correspondence.

First, RS data were segmented by the sliding-window approach. For each subject, T = 192 TR was divided by L = 7 windows. Therefore, each window contained t = 48 TRs (window size of 96 s), and 50% of time points overlapped between two sequential windows (Figure 1).

Next, the images within each time window were reshaped into a matrix (T by V, where V is the number of voxels within whole brain), denoted by a vector X^[m,l]^, m = 1,…, M; l = 1,…, L. Voxels were regarded as samples for each time point. The total number of subjects M was 24. Therefore, we performed joint blind source separation of these M × L data sets, each of size T × V.

Thirdly, IVA was utilized to achieve joint blind source separation and SDC was extracted from multiple subjects and different time windows concurrently. Supposing each data set X^[m,l]^ from the mth subject at the lth window is formed by the linear mixtures of N independent sources:


$$x^{\left[m,l\right]}=A^{\left[m,l\right]}\;s^{\left[m,l\right]}\;m=1,\ldots ,M;l=1,\ldots ,L$$

Where s^[m,l]^ = [s_1,m,l_,…,s_n,m,l_,…, s_N,m,l_]^T^ is the SDC vector of size N × V; element s_n,m,l_ is the n-th underlying component and A^[m,l]^ is a T × N mixing matrix with the n-th column representing the time course associated with the n-th SDC. To represent associated SDCs across all subjects, the n-th SCV was constructed by taking each n-th SDC from all subjects and windows, i.e., $s_{n}=[s_{n,1,1},\ldots ,s_{n,1,L},\ldots ,s_{n,M,1},\ldots ,s_{n,M,L}],n=1,\ldots ,N$

The goal of IVA is to determine M × L demixing matrices w^[m,l]^ and the corresponding SDC vector estimates y^[m,l]^ = W^[m,l] [m,l]^, m = 1,…, M; I = 1,…, L. The estimated SCVs, defined as y_n_ = [y_n,1,1_,…,y_n, M, L_]^T^, n = 1,…, N are maximally independent from each other, where y_n,m,l_ = (W_n,m,l_)^T^ x^[m,l]^; (W_n,m,l_) is the nth row of W^[m,l]^. IVA decomposition can be achieved by minimizing the mutual information (MI) (38) among SCVs.

In this study, IVA implementation, namely IVA-GL, was incorporated in the GIFT toolbox. This took advantage of IVA-G (multivariate Gaussian distribution) and IVA-L (multivariate Laplacian distribution) and IVA-L was initialized with a solution from IVA-G to achieve IVA-GL decomposition. IVA-GL can yield more robust joint blind source separation than IVA-L or IVA-G alone.

##### 2.3.2.2 SDC analysis

After SDC estimation, spatial dynamic FNC among these SDCs were quantified by MI. For SDCs in one window, the subject’s MI values between two SDCs would be calculated as SDC connectivity. A normalized measure of SDC connectivity was obtained, in the range of (0, 1), with a value of zero meaning complete independence. The standard deviation (STD) of the normalized MI between two SDCs for all windows and all subjects of both groups was calculated to compare both groups.

### 2.4 Statistical and correlation analyses

Statistical analysis was carried out with the GIFT toolbox.

#### 2.4.1 Temporal static and dynamic FNC

To examine the distribution of brain network FC in the whole time series, two-sample t-test was performed to compare the TS and N groups for static FNC strength, dynamic FNC strength and duration of each connection state. Because the durations of the connection state in various subjects differed, a patient was deemed not to belong to this state with less than 10 windows of a state. The FDR test was performed, and *p* < 0.05 was considered statistically significant.

#### 2.4.2 Connectivity of SDC and spatial dynamic FNC

For each window, the spatial connectivity between all possible pairs of SDCs was determined. The STD of FC across windows was obtained for each patient. Two-sample t-test was utilized to assess whether the FC within each time window significantly differed between the TS and N groups. The Mann–Whitney U-test was performed to assess whether the STD of FC was significantly different between the TS and N groups. The FDR test was carried out, and *p* < 0.05 was considered statistically significant.

#### 2.4.3 Correlation analysis of differences and TS clinical indicators

Correlation analysis was performed to examine the relationship between the clinical indices (YGTTS score and disease duration) and the fMRI metrics that exhibited statistically significant group differences. Pearson correlation coefficients were calculated, and a *p*-value < 0.05 was considered statistically significant.

## 3 Results

### 3.1 Analysis of the temporal domain

#### 3.1.1 Identified temporal domain components

**Figure 2:**
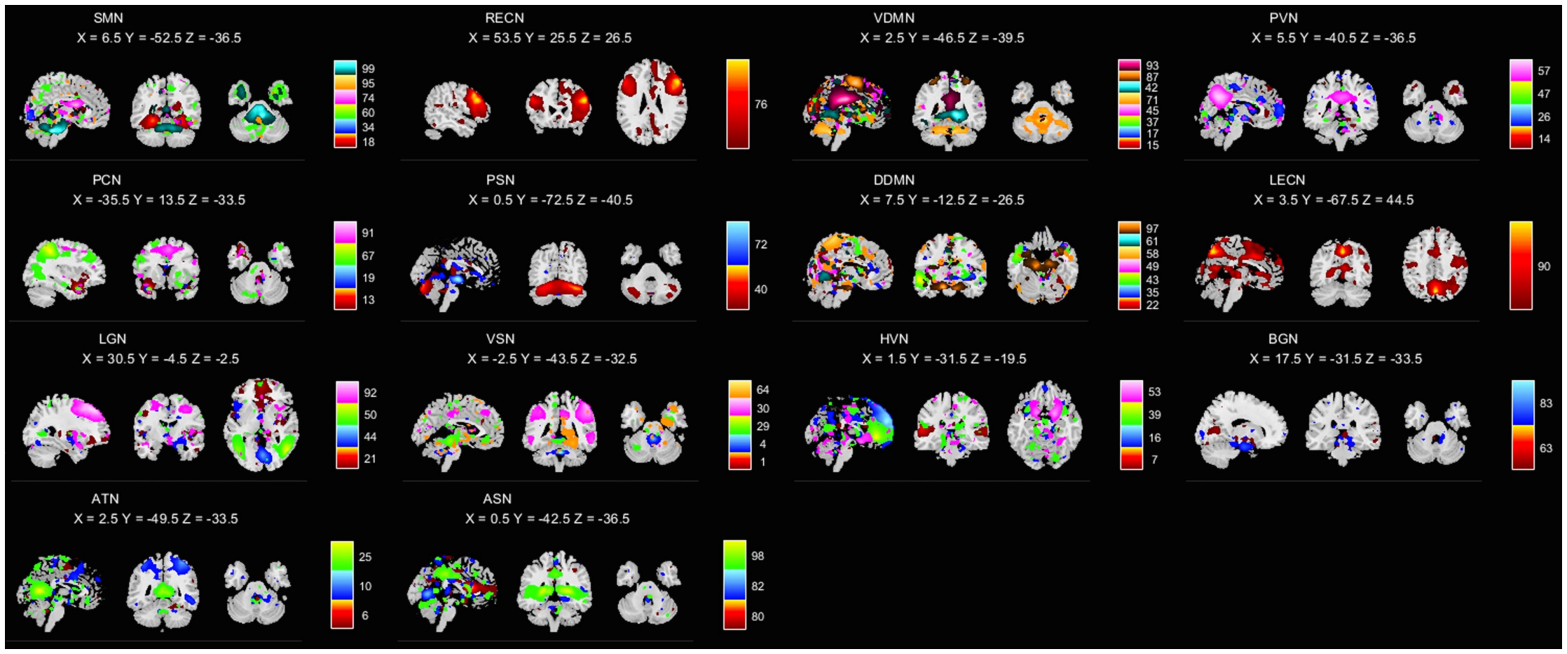
Identified TDCs. Within each ICN, the color of the components corresponds to no. of TDCs. X, Y, Z correspond to the MNI coordinates.

Totally 100 TDCs were identified by GICA to define brain networks. After removing noise, 54 TDCs were identified as 14 intrinsic connectivity networks (ICNs). The detailed features of the identified networks are shown in Table 1. The distributions of TDCs are shown in Figure 2.

**Table 1:** Identified TDCs.

Name of ICN	Abbreviation of ICN	No. of TDC (Correlation with RSN template)
Sensorimotor Network	SMN	18(46%), 34(38%), 60(20%), 74(34%), 95(20%), 99(25%)
Right Executive Control Network	RECN	76(26%)
Ventral Default Mode Network	VDMN	15(20%), 17(50%), 37(21%), 42(23%), 45(46%), 71(64%), 87(20%), 93(20%)
Primary Visual Network	PVN	14 (22%), 26(31%), 47(41%), 57(44%)
Precuneus Network	PCN	13(29%), 19(54%), 67(24%), 91(30%)
Posterior Salience Network	PSN	40(23%), 72(20%)
Dorsal Default Mode Network	DDMN	22(45%), 35(49%), 43(26%), 49(20%), 58(41%), 61(36%), 97(27%)
Left Executive Control Network	LECN	90(22.23)
Language Network	LGN	21(31%), 44(36%), 50(31%), 92(25%)
Visuospatial Network	VSN	1(35%), 4(21%), 29(31%), 30(20%), 64(28%)
Higher Visual Network	HVN	7(30%), 16(33%), 39(29%), 53(23%)
Basal Ganglia Network	BGN	63(22%), 83(34%)
Auditory Network	ATN	6(35%), 10(37%), 25(38%),
Anterior Salience Network	ASN	80(31%), 82(25%), 98(24%)

The value in the parenthesis indicates correlation value between TDCs and RSN template.

#### 3.1.2 Static FNC

Static FNC showed no statistically significant difference between the two groups.

#### 3.1.3 Dynamic FNC

Dynamic FNC in all subjects could be clustered into three states. There was no statistically significant difference between the two groups in terms of dwell time in windows for each state.

**Figure 3:**
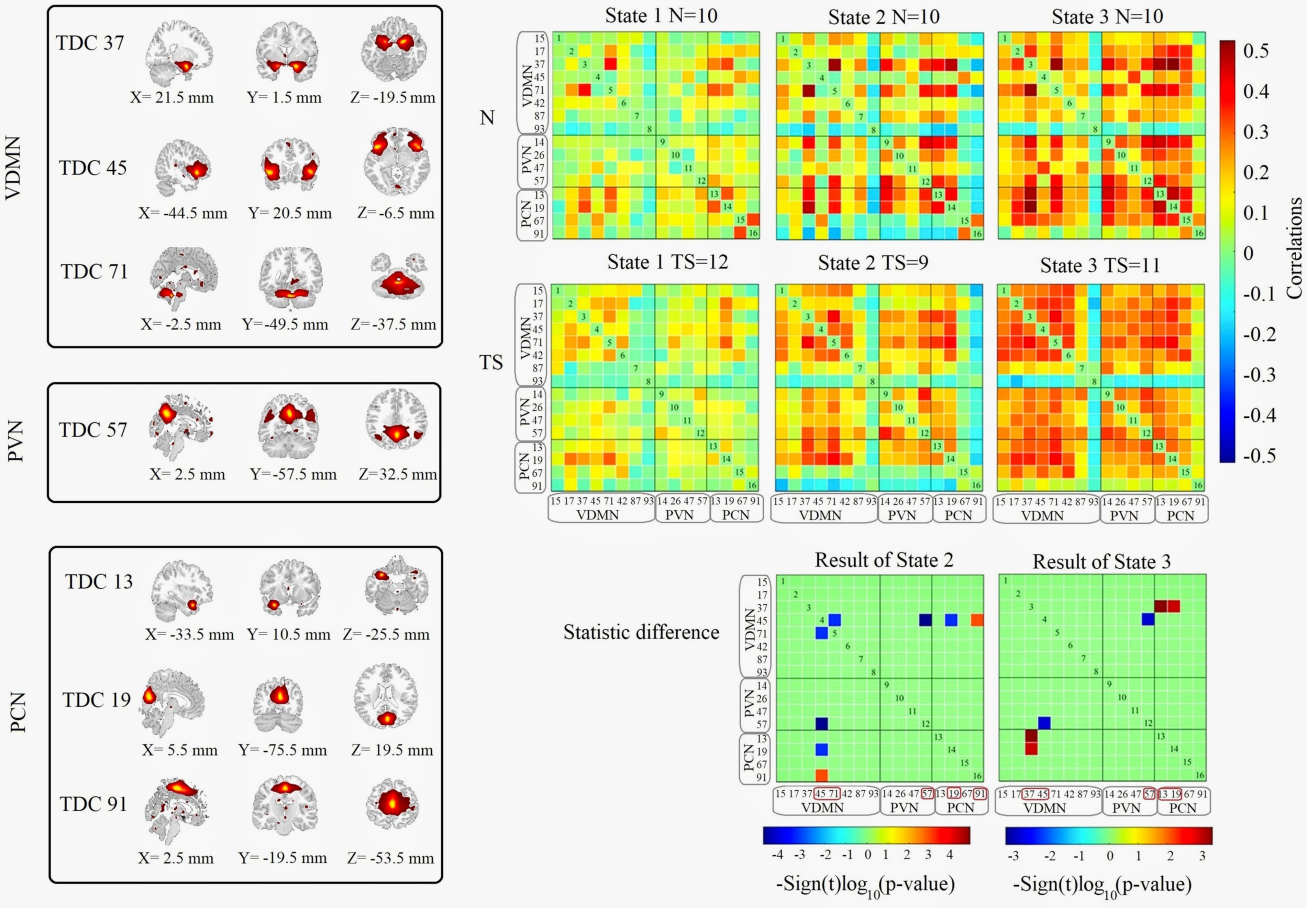
The result of statistically significant differences in dynamic FNC. Left panels shows the location of the TDC. Right panels are display Pearson correlation of two groups in 3 states. Total number of subjects in each state are list above each picture. Statistically significant differences in dynamic FNC between all networks are computed for each of the 3 states using with 2-sample *t*-tests at a significance level of *q* < 0.05, corrected by FDR. Networks with statistically significant differences are show in this figure and effects are color-coded for *p*-value.

Dynamic FNC for the Ventral Default Mode Network (VDMN), Precuneus Network (PCN) and Primary Visual Network (PVN) in States 2 and 3 had statistically significant differences between the two groups (Figure 3). In State 2, statistically significant differences were found in the internal connections of VDMN (TDC 45, corresponding to the Insula, & TDC 71) and the connections between VDMN (TDC 45) and PCN (TDC 57) and PVN (TDC 19, TDC 91). In State 3, statistically significant differences were found between VDMN (TDC 37, 45) and PVN (TDC 57) and PCN (TDC 13, TDC 19).

### 3.2 Analysis of spatial domains

#### 3.2.1 Identified SDC

**Figure 4:**
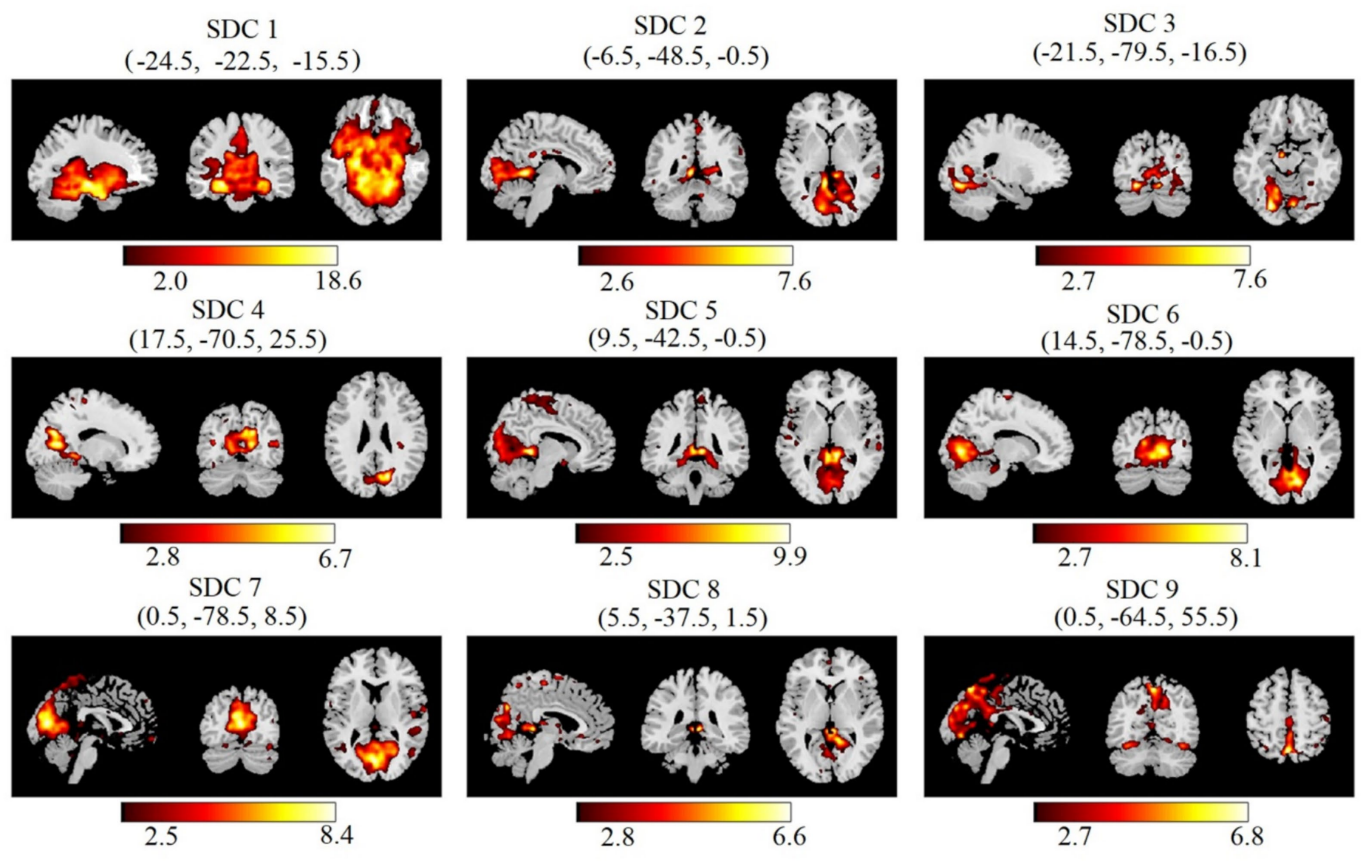
Nine SDCs of interest derived from IVA decomposition. These are MNI coordinates in parentheses.

The 192 time points for each patient were divided into 7 windows and 20 reliable estimated components. We visually inspected the spatial maps and removed the components associated with artifacts. Finally, nine SDCs of interest for each subject and each window were selected (Figure 4).

#### 3.2.2 Spatial dynamic FNC

**Figure 5:**
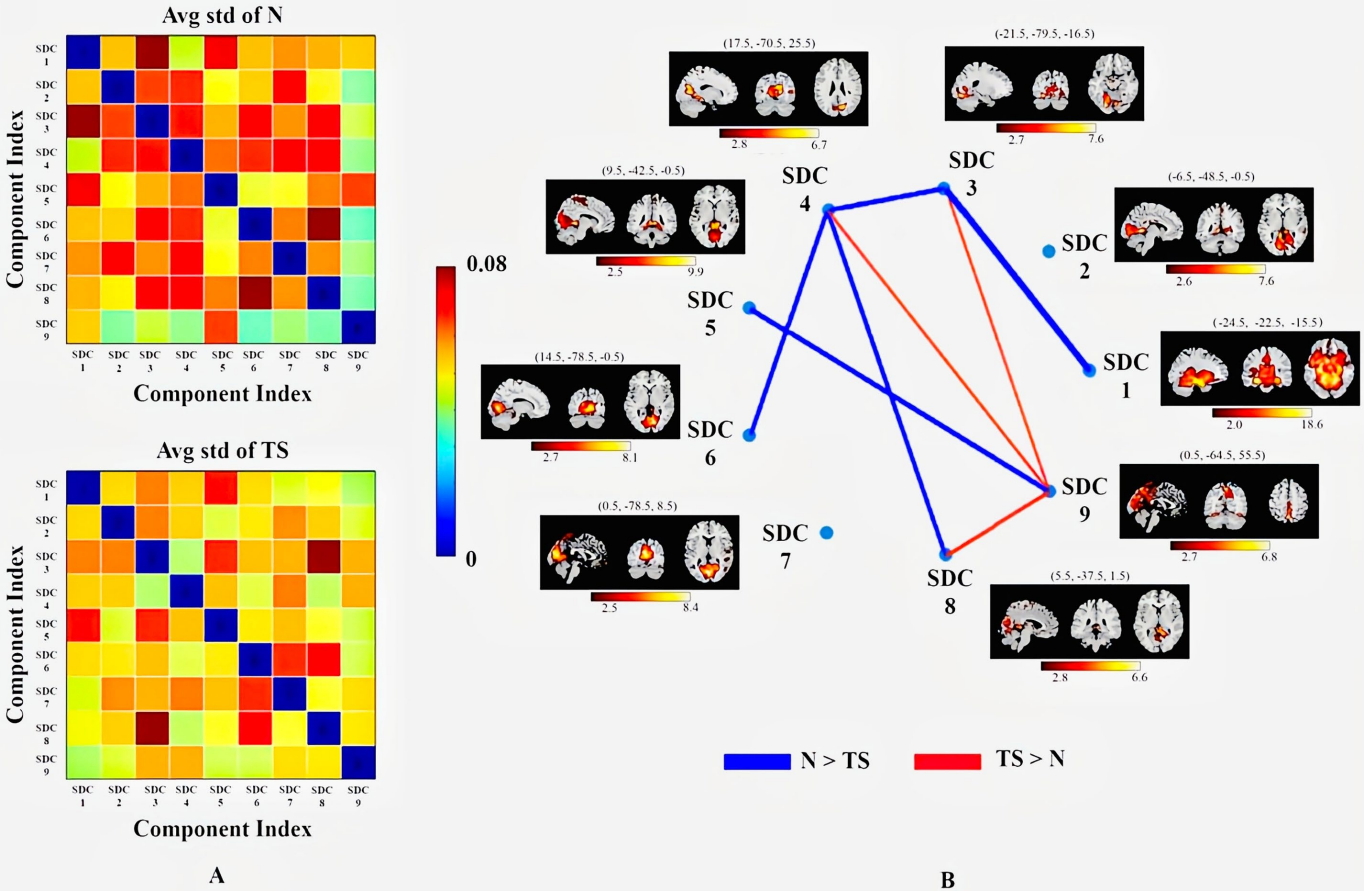
Statistical metrics quantifying connectivity dynamics. **(A)** Matrices of the average STD of normalized MI for the healthy control (N) group (top) and TS group (bottom). The color bar indicates STD magnitude. **(B)** Mann–Whitney *U*-test of each connectivity STD between the N and TS groups (*p* < 0.1). The results displayed show marginal differences at a threshold of *p* < 0.1 (FDR corrected). The SDCs are identified and shown as in Figure 4. The thicker the line, the more significant is the median value of connectivity changes over time.

The average value of STD for each connection point over 7 time windows and all subjects in each group were determined (Figure 5A). The redder the color, the higher the variability at that connection point. The pairs of SDCs had marginal group differences in connectivity’s STD (Figure 5B).

### 3.3 Associations of differences in the above results with YGTSS score and disease duration

YGTSS score was negatively correlated with the MI of SDC 5 (corresponding to the parahippocampal gyrus and amygdala) ~ SDC 9 (precuneus), with a correlation coefficient of 0.705. The difference in the MI of SDC 5 ~ SDC 9 between the two groups decreased with increasing YGTSS score. Suggesting that lower connectivity strength in these components is associated with more severe clinical symptoms.

The duration of disease was positively correlated with Pearson correlation coefficients for TDC 45 ~ TDC 71 and TDC 45 ~ TDC 19 in State 2, and correlation coefficients were 0.698 and 0.787, respectively. The difference of Pearson correlation coefficients for TDC 45 ~ TDC 71 and TDC 45 ~ TDC 19 in State 2 between the two groups increased with the duration of TS.

## 4 Discussion

### 4.1 Time and spatial dynamic functional network connections

Human brain activities are dynamic in nature, and thus, dynamic connectivity analysis is an insightful tool to investigate instantaneous changes (39); the enriched information conferred by the analysis might better reflect the temporally fluctuating brain state compared with static connectivity analysis as shown in previous studies (14, 40). Recent studies have extended this approach to TS, revealing altered temporal stability, reduced network flexibility, and abnormal transitions among functional states in both children and adults with TS (41–43). In this work, there were statistically significant differences in temporal dynamic FNC values and marginal differences in spatial dynamic FNC values.

In time dynamic FNC analysis, the entire time series was clustered into 3 states. As shown in Figure 3, statistically significant differences were found in states 2 and 3 at VDMN, PVN and PCN. With the progression of state 1 to state 3, in the N group, Pearson correlation coefficients between select TDCs became larger and some of them became smaller or unchanged, showing obvious strength and weakness regularity; in the TS group, Pearson correlation coefficients between TDCs did not increase significantly, but the number of connections of TDCs increased significantly. This suggests that although subjects in the TS group exhibited a greater number of functional brain connections, they lacked the ability to strengthen connections between relevant brain regions.

In spatial dynamic FNC analysis, totally 9 effective components have been identified by spatial dynamic network and all those belong to 5 networks, i.e., SMN, PVN, PCN, DDMN and VDMN. The results of FC analysis in the entire time series are shown in Figure 4, and statistically significant differences between the two groups were found in PVN and VDMN. The average KLD fluctuated more significantly in the N group than in the TS group. This inferred that the changes of connectivity strength were more obvious in the normal group compared with the TS group. These results align with recent evidence that the TS brain exhibits decreased temporal variability of FC and less frequent transitions between functional states, reflecting constrained dynamic reorganization (41, 42).

Combining the obtained time and spatial dynamic FNC data, statistically marginal differences were found in three networks: the ventral default network (VDMN) was part of the default mode network (DMN). The range of FNC strength in the N group was larger and the connection strength was higher. DMN showed activation in the resting state, and deactivation during goal-oriented behavior. Recent studies have shown that the DMN plays an important role in internally-oriented mental processes, and different regions within it are responsible for different tasks. The VDMN was thought to be activated when subjects are focused on their internal mental-state processes, including self-referential processing, interception, autobiographical memory retrieval, and imagining future (44). The PCN is adjacent to and independent of the default mode network, which was determined to be involved in the memory recall process (45). The spontaneous activity patterns of PVN are significantly associated with visual mental imagery processes in the resting brain (46). Disrupted dynamics among these networks may therefore underlie impaired self-monitoring and inhibitory control in TS (43). A resting-state fMRI study confirmed that reduced temporal correlation coefficients (tCC) and unstable network transitions in cortico-basal ganglia-cerebellar circuits strongly correlate with tic severity, consistent with our observations (41).

### 4.2 Associations of differences in the above results with YGTSS score, duration of disease

High scores were negatively correlated with most of the differences, mainly involving the parahippocampal gyrus, amygdala and precuneus. Because the subjects in this study were young children at the early stage of the disease, it is speculated that YGTSS scores might be insufficient in assessing physiological changes of patients in early diagnosis. Therefore, it was suggested that multiple periodic follow-up evaluations should be performed in the later period to improve the accuracy of disease diagnosis.

Long duration of disease was positively correlated with most different regions, indicating that the biomarkers explored in this study can effectively reflect the duration of the disease. The correlation between TDC 45 network connection in state 2 and disease duration was relatively high. As shown above, the TDC 45 contains the insula. With disease duration, the connections between the insula and other networks increased in the TS group during sleep. This mirrors findings from longitudinal neuroimaging, indicating adaptive reorganization of cortico-striato-thalamo-cerebellar circuits in chronic TS (42, 47). The heightened insula co-activation may reflect elevated arousal and interoceptive awareness, known to modulate tic expression and daytime behavior (48–52).

### 4.3 Involved brain regions of FNC

To summarize the abovementioned results, temporal and spatial analyses collectively revealed group differences in regions including the superior temporal gyrus, parahippocampal gyrus, amygdala, inferior parietal, lingual gyrus, insula, cuneus, calcarine fissure and surrounding cortex (calcarine), inferior frontal gyrus, middle frontal gyrus (orbital part), SMA, posterior cingulate gyrus, precuneus and paracentral lobule. It is important to note that while the temporal dynamic analysis yielded statistically significant differences, the spatial dynamic analysis primarily indicated marginal trends in these regions. These findings generally corroborated previous studies (48, 49), and are further supported by recent multimodal meta-analyses (42, 43). In state 2 of the temporal dynamic FNC, Pearson correlation coefficients for TDC 45 (VDMN) and TDC 71 (VDMN), TDC 57 (PVN), and TDC 19 (PCN) in the TS group were significantly increased, and the significant increase was strongly positively correlated with disease duration. The comparison of functional connections between the two groups showed weak connections between TDC 45 and other components in the N group, while strong connections were found in the TS group. The regions of TDC 45 involved the insula, which is a cytoarchitectonic complex and a richly connected structure that functions as a cortical hub involved in interoception, multimodal sensory processing, autonomic control, perceptual self-awareness, and emotional guidance of social behavior (50). Subjects in this study were asleep, and it was inferred that the insula was not activated, which may be the reason why the Pearson coefficients between TDC45 and other components were low in the N group and high in the TS group. It was suggested that increased insula coactivation with brain networks is associated with arousal (51). There is evidence that children with TS have disturbed sleep quality and increased arousal, both of which may be intrinsic to the disorder and might trigger tics and other behavioral problems during daytime (52). Motor inhibition and self-regulation hubs, including SMA, inferior frontal gyrus, and posterior cingulate, also showed weakened coupling in TS. This aligns with previous dynamic FC findings, implicating SMA-frontal circuits in impaired tic suppression (53). Recent network-level studies highlight thalamo-frontal and cingulo-opercular circuits as central to tic generation and suppression. Connectivity between DBS targets and these networks predicts tic improvement (54), while alpha-band thalamo-frontal connectivity inversely relates to symptom severity (55). Together, these results indicate that structural and dynamic functional disorganization within cortico-striato-thalamo-cortical loops underlies TS pathophysiology.

## 5 Conclusion

Twenty-four subjects aged 4–6 years were recruited to participate in this fMRI study of TS. Time and spatial dynamic functional network connections were analyzed. The results revealed statistically significant differences in temporal dynamic FNC values (specifically within States 2 and 3) and marginal differences in spatial dynamic FNC values, mainly involving the VDMN, PCN, and PVN. The N group exhibited a broader dynamic range and higher overall magnitude of FNC strength compared to the TS group. Conversely, the TS group demonstrated abnormally elevated connectivity specifically within insula-related components. These indexes strongly positively correlated with disease duration and might be the reason for triggered tics during daytime. This study suggested differences in brain networks in early stages of the disease in young children and provided a basis for exploring early biomarkers of TS.

## Data Availability

The original contributions presented in the study are included in the article/supplementary material, further inquiries can be directed to the corresponding author.

## Footnotes

1
https://www.fil.ion.ucl.ac.uk/spm/software/spm12/

2
http://mialab.mrn.org/software/gift/index.html
